# Retrospective Review of Pectoralis Major Ruptures in Rodeo Steer Wrestlers

**DOI:** 10.1155/2013/987910

**Published:** 2013-06-06

**Authors:** Breda H. F. Lau, Dale J. Butterwick, Mark R. Lafave, Nicholas G. Mohtadi

**Affiliations:** ^1^Department of Physical Education and Recreational Studies, Mount Royal University, 4825 Mount Royal Gate SW, Calgary, AB, Canada T3E 6K6; ^2^Faculty of Kinesiology, University of Calgary, 2500 University Dr NW, Calgary, AB, Canada T2N 1N4; ^3^Faculty of Medicine, Health Sciences Centre, Foothills Campus, University of Calgary, 3330 Hospital Drive NW, Calgary, AB, Canada T2N 4N1

## Abstract

*Background*. Pectoralis major tendon ruptures have been reported in the literature as occupational injuries, accidental injuries, and sporting activities. Few cases have been reported with respect to rodeo activities. *Purpose*. To describe a series of PM tendon ruptures in professional steer wrestlers. *Study Design*. Case series, level of evidence, 4. *Methods*. A retrospective analysis of PM ruptures in a steer wrestling cohort was performed. Injury data between 1992 and 2008 were reviewed using medical records from the University of Calgary Sport Medicine Center. *Results*. Nine cases of pectoralis major ruptures in professional steer wrestlers were identified. Injuries occurred during the throwing phase of the steer or while breaking a fall. All athletes reported unexpected or abnormal behavior of the steer that contributed to the mechanism of injury. Seven cases were surgically repaired, while two cases opted for nonsurgical intervention. Eight cases reported successful return to competition following the injury. *Conclusion*. Steer wrestlers represent a unique cohort of PM rupture case studies. Steer wrestling is a demanding sport that involves throwing maneuvers that may predispose the muscle to rupture. All cases demonstrated good functional outcomes regardless of surgical or non-surgical treatment.

## 1. Introduction

Pectoralis major (PM) tendon ruptures have been reported in the literature. These ruptures present as occupational injuries [1, 2], accidental injuries [3], and sporting activities [4–7] primarily in weight lifting. Ruptures have also been reported in elderly patients during procedures for transferring, positioning, and dressing the patients [8–10]. Ruptures of the muscle belly typically present as the result of direct trauma or traction injuries. Falls onto outstretched arms often result in ruptures at the musculotendinous junction, whereas excessive tension on a maximally contracted muscle often results in ruptures of the tendinous insertion from the humerus [6, 8]. Sport-related PM ruptures usually result from activities requiring a large amount of upper body strength. Two articles have reported PM ruptures resulting from a rodeo event: one reported a professional bull rider who tore his left PM during a bull riding [11]; the other reported three cases of PM ruptures during steer wrestling but failed to describe the events surrounding the injuries [12]. These three cases are included in this study and reported in further detail. The purpose of this paper is to describe a series of PM ruptures in professional rodeo steer wrestlers in order to expand upon and understand the nature of this injury behind this particular cohort of athletes. Therefore, descriptive information was gathered about the sport to identify prevalence, characterize PM ruptures, and determine mechanism of injury affecting rupture type and location. 

 Each year professional rodeo contestants compete during the rodeo season in an attempt to qualify for the national championship rodeo in Canada, the Canadian Finals Rodeo. Steer wrestling is one of the seven events at these finals. Steer wrestling is a rodeo event that requires considerable strength in addition to timing and coordination. The event consists of a 450 to 750 lb steer and two mounted cowboys: the contestant and the hazer. The steer is placed into a chute, with the contestant and the hazer on either side of the chute. The contestant waits behind a rope fastened to a string that is attached to the chute. When the contestant is ready, he calls for the steer, and the chute opens. The steer is given a head start, and the contestant must catch up to the steer. The job of the hazer is to mimic the pathway of the contestant on the opposite side of the steer relative to the contestant, in an effort to keep the steer running as straight as possible. The goal of the contestant is to wrestle the steer to the ground in a timely fashion. 

 There are three phases to steer wrestling: (1) the dismount: when the cowboy transfers from his horse to the steer; (2) the slide: when the cowboy positions himself for the throw; and (3) the throw: when the steer is wrestled to the ground with all four legs and head facing in the same direction [13]. The cowboy must position himself in a manner that allows his right arm to reach under the steer's right horn and his left hand to grab the top of the steer's left horn. As the cowboy dismounts from his horse, he uses his legs to brace against the ground, generating a large deceleration force. He must then lower his center of gravity and sit back. Finally, the cowboy must flex, adduct, internally rotate, and concentrically contract his right arm, while internally rotating the steer's right horn in an attempt to turn and tackle the steer down onto its left side. 

## 2. Materials and Methods

Patient records from the University of Calgary Sport Medicine Center were retrospectively reviewed from 1992 to 2008. Patients were identified through diagnostic codes searching for PM injuries. To ensure that cases were not overlooked, cases were also searched using anatomical location (i.e., shoulder). Cases were also identified from caregivers of the Canadian Pro Rodeo Sport Medicine Team (CPRSMT). All patients diagnosed with a PM injury were reviewed. Clinical assessments were confirmed by a sport medicine physician, orthopaedic surgeon, or athletic therapist. Patient medical records and retrospective interview were used to collect the following information: patient demographics, previous medical history, mechanism of injury, physical findings, diagnostic imaging, treatment, and treatment outcome. This project was approved by the University of Calgary Conjoint Health Research Ethics Board. 

## 3. Results

 During the years 1992 to 2008, a total of 34 patients with a PM muscle injury were treated at the University of Calgary Sport Medicine Center. Six PM ruptures were reported in rodeo athletes, five of which were in steer wrestlers. Four additional cases of PM ruptures not treated at the center were identified by caregivers of the CPRSMT. In total, there were nine professional male steer wrestlers identified with PM ruptures of the right arm at the distal insertion of the PM into the humerus between 1992 and 2008. A complete tear of the PM was determined upon clinical examination of the injury presenting with discontinuity of the tendon at the musculotendinous junction or near insertion into the humerus and loss of muscular contour. The patient's ages ranged from 30 to 51 years (mean: 35 ± 7 years). Incomplete ruptures of the PM were also identified but were not included in this study. All cases occurred during rodeo competition participating at Canadian Professional Rodeo Association sanctioned rodeos. 

Four of nine cases (44.4%, 95% CI: 12.0–76.9%) had a previous history of the right shoulder injury including rotator cuff tendinopathy, glenohumeral dislocation, and biceps brachii strain. Four cases (44.4%, 95% CI: 12.0–76.9%) reported previous PM strains. Three cases did not have previous injuries to either the right shoulder or PM (33.3%, 95% CI: 2.5–64.1%). 

### 3.1. Mechanism of Injury

In seven of nine cases (77.8%, 95% CI: 50.6–104.9%) the PM was injured during the attempted throwing phase of the steer. For the other two cases (22.2%, 95% CI: −4.9–49.4%), injuries resulted when the contestant was forced to break his fall on the ground with his arm abducted and extended out to one side.

Table 1 presents trends surrounding events leading up to the incident that may have contributed to the mechanism of injury during the steer wrestling activity (e.g., steer behavior, horse behavior, hazer error). In all nine cases, unexpected or abnormal behavior of the steer was identified as a potential risk factor. In four cases (44.4%, 95% CI: 12.0–76.9%), the steer stopped prematurely during the chase. In three cases, both the steer and the hazer veered wide (33.3%, 95% CI: 2.5–64.1%). In two cases (22.2%, 95% CI: −4.9–49.4%), the mechanism of injury was due to unexpected movement of the steer as it failed to set up properly either by slowing down or bucking upwards. 

### 3.2. Physical Examination and Imaging

 Clinical examination revealed that eight of nine cases (88.9%, 95% CI: 68.3–109.4%) presented with full range of motion in the shoulder. One case was limited in external rotation and abduction but had sustained previous partial ruptures (*n* = 2) to the same PM. One case reported transient numbness in the hand distal to the injury site. The cases presented with physical findings including pain, swelling, ecchymosis, obvious defect due to muscle retraction, and weakness in resisted internal rotation, adduction, and horizontal flexion of the shoulder. Plain radiographs were ordered in three cases (33.3%, 95% CI: 2.5–64.1%), and one case received both ultrasound and magnetic resonance imaging. 

### 3.3. Operative versus Nonoperative Treatment

Of nine cases, only two cases (22.2%, 95% CI: −4.9–49.4%) elected nonsurgical treatment with conservative progressive rehabilitative exercises. Eight of nine cases (88.9%, 95% CI: 68.3–109.4%) were able to successfully return to participation in professional steer wrestling. Both surgical and nonsurgical patients received physician directed rehabilitation and return to play guidelines. Rehabilitation included progressive exercises focusing on improving range of motion, strength, and endurance. One patient subsequently won the Canadian Finals Rodeo. 

## 4. Discussion

The first documented PM rupture case dates back to 1822, and since then, there have been more than 200 cases reported in the literature [14]. This report presents the largest series of ruptured PM tendons reported during steer wrestling. A five-year epidemiological study of injuries in Canadian professional rodeo indicated that steer wrestlers sustained 9.2 injuries/1000 steer wrestling exposures [15]. In the same study, the shoulder complex was the second most frequently injured body part when considering all rodeo events as a whole. Complete ruptures of muscles, tendons, or ligaments constituted 7% of all injuries. Rodeo caregivers found that distal biceps tendon ruptures in bull riders and some bareback riders, as well as complete ruptures of the PM [16] or latissimus dorsi [17, 18], were significant career interrupting events for these athletes. 

The physical findings from these cases support diagnostic findings in the literature. Patients often experience a tearing sensation presenting with a large hematoma noted on acute evaluation. This is accompanied by limited shoulder motion secondary to pain and weakness on manual muscle testing for resistance to adduction or internal rotation [19]. All nine cases presented with a combination of the following signs or symptoms: tearing sensation, palpable or visible defect against isometric resistance, ecchymosis, swelling, pain, weakness, decreased range of motion, and decreased strength.

Two patients continued to steer wrestle without surgical intervention and one continued to steer wrestle during a national finals rodeo for six steer wrestling attempts after which surgical repair was completed. Although other internal rotators and adductors of the shoulder can apply forces to these movements, we found no reports of isokinetic or eccentric testing of these movements following PM rupture. Despite other muscles compensating, it is unlikely that there were no overall strength deficits. 

 The PM is a powerful adductor and internal rotator of the arm. Additionally, it contributes to forward flexion when the arm is in the extended or neutral position and backward extension when the arm is in the forward flexed position. The clavicular portion of the muscle serves primarily to forward flex and adduct the arm, whereas the sternocostal portion serves primarily to internally rotate and adduct the arm. The PM is at risk during any activity in which the arm is extended and externally rotated while under maximal contraction [20]. For example, when PM injuries occur during weight training, the typical mechanism of injury is excessive tension from acute overload of an eccentrically loaded tendon. Fibers originating from the sternal head are maximally stretched when the weight is eccentrically lowered to the chest during bench press. During the deceleration and early lift phase of this exercise, the fibers can be overstretched, leading to failure of the viscoelastic force and rupture [11].

During the throwing phase in steer wrestling, the PM concentrically contracts to adduct the shoulder across the body in an attempt to throw the steer to the ground. Several steer wrestlers indicated that the steer stopped, veered away from the athlete, or bucked upwards. These unexpected steer movements instead resulted in a sudden, powerful eccentric load on the PM causing seven cases of complete ruptures of the PM at the humeral insertion of the right arm. It is not surprising that all PM ruptures occurred to the right arm as this is the arm that all steer wrestlers use to catch the horn of the steer in order to lever the steer to the ground. 

Further risk factors to tendon rupture include muscle fatigue and microtrauma [20]. Six of nine cases in this study suffered previous injuries to the right shoulder or arm. 

Steer wrestling is a demanding sport that involves a throwing maneuver that places the PM in an unusually heavy and eccentric load which may predispose the PM to rupture. It is acknowledged that independent, nomadic lifestyles of professional rodeo athletes present challenges to injury management, treatment, and prevention. For instance, only four contestants reported maintaining a structured strength training and flexibility program prior to injury. One study has suggested that tendons may not be able to adjust to increasing muscle mass and decreased flexibility associated with weight training [21]. This case series review illustrates that there may be advantages for structured, prospective study of PM ruptures in steer wrestling. 

## 5. Conclusions

Canadian steer wrestlers present a unique cohort of PM ruptures. These cases demonstrate functional successful outcome with a variety of treatments. Treatment and rehabilitation decisions appear to lack evidence-based decision making. Rodeo is a popular international sport that takes place in countries such as the United States of America, Brazil, and Australia. We suspect that this particular injury is not unique to Canadian steer wrestlers. Therefore, this case series may serve as a future stimulus for an international structured approach examining prevention, treatment, rehabilitation, and return to participation. 

## Figures and Tables

**Table 1:** Demographic data for all nine injured steer wrestlers.

Case	Age	Prior shoulder injury	Prior PM* injury	Mechanism	Source of error
1	30	No	No	Throw	Steer bucked upwards unexpectedly
2	30	Bicep brachii strain	No	Fall	Hazer and steer veered wide
3	31	No	Yes	Fall	Steer stopped
4	32	GH** dislocation	No	Throw	Steer veered wide
5	33	RC tendinopathy	Yes	Throw	Steer and hazer veered wide
6	33	No	No	Throw	Steer stopped
7	33	No	No	Throw	Steer stopped
8	48	RC^*+*^ tendinopathy	Yes	Throw	Steer stopped
9	50	No	Yes	Throw	Steer slowed down

*PM: pectoralis major; **GH: glenohumeral; ^*+*^RC: rotator cuff.
